# Stability of treatment with self-ligating brackets and conventional brackets in adolescents: a long-term follow-up retrospective study

**DOI:** 10.1186/1746-160X-10-41

**Published:** 2014-09-20

**Authors:** Zhou Yu, Lin Jiaqiang, Chen Weiting, Yi Wang, MinLing Zhen, Zhenyu Ni

**Affiliations:** DDS, Hospital of Stomatology, Wenzhou Medical University, Wenzhou, China; Department of Orthodontics, Hospital of Stomatology, Wenzhou Medical University, Wenzhou,113 west college road, 325000 Wenzhou, China

**Keywords:** Self-ligating brackets, Conventional brackets, Stability

## Abstract

**Objectives:**

The aim of this study was to assess the long-term stability of treatment with self-ligating brackets compared with conventional brackets.

**Materials and methods:**

The long-term follow-up retrospective study sample consisted of two groups of patients: group SL (including passive and interactive self ligating braces) comprised 30 subjects treated with self-ligating brackets at a mean pretreatment (T0) age of 13.56 years, with a mean follow up period for 7.24 years; group CL comprised 30 subjects treated with conventional brackets at a mean pretreatment age of 13.48 years, with a mean follow up period for 7.68 years. Relapse were evaluated by dental casts examination using the Peer Assessment Rating (PAR) index and the Little irregularity index. The two groups were evaluated for differences in the changing of PAR and Little irregularity index using paired-t tests. Inter-observer and intra-observer reliability was assessed by means of the Pearson’s correlation coefficients method.

**Results:**

There were no significant differences changed in PAR and the Little irregularity index between groups for the long-term follow-up period.

**Conclusions:**

The study revealed that brackets type did not affect the long-term stability. Considering self-ligating brackets were expensive, given comprehensive consideration for the patients to choose suitable orthodontic bracket type was of critical importance.

## Introduction

The stability of aligned teeth is variable and unpredictable [1]. Therefore, the maintenance of dental alignment after orthodontic treatment is considered to be a challenge for the orthodontics. Follow-up studies of treated cases have shown that although improvement in the dentition can obviously be achieved, there is a tendency of relapse many years after treatment [2, 3]. The reason is complex, several factors may account for this relapse, including inter-canine width [4], mandibular growth rotation [5], third molar eruption [6], influence of gingival tissues [7], or treatment modalities [8]. Some investigators claim that lower force produced by self ligating bracket systems might result in more physiological tooth movement, therefore, SLBs produce more stable treatment results [9].

Self-ligation bracket is not a new concept. This brace system has undergone a renaissance over the past 20 years with enhanced ingenuity and reliability [10]. According to the ligating mechanisms, self-ligating brackets can be divided into 2 main categories, active and passive self-ligating brackets. Active self-ligating brackets have a spring clip which press against the arch-wire for better control of rotation and torque. Conversely, passive self-ligating brackets usually have a slide that press no active force on the arch-wire.

Many advantages of self-ligating bracket system have been claimed, including reduced friction [11], more efficient tooth movement, less treatment time, increased patients acceptance, and superior treatment results [12, 13]. Unfortunately, a systematic review [14] claimed that there was lack of significant overall effects apparent in this meta-analysis contradicts evidence-based statements on the advantages of self-ligating brackets over conventional ones regarding discomfort during initial orthodontic therapy, number of appointments, and total treatment time. Besides, a recent systematic review of self-ligating bracket stated [9] that, at this time, no studies comparing the stability of treatment result with self-ligating brackets to conventional brackets were identified.

Therefore, the objective of this study was to assess the long-term stability of treatment with self-ligating brackets compared with conventional brackets.

## Materials and methods

### Subjects

This research was approved by the Ethics Committee of the WenZhou Medical University.

The sample size for each group was calculated based on an alpha significance level of 0.05 and a beta of 0.1 to achieve 90% power to detect a clinically meaningful difference of two (PAR/IR) between the self-ligating group (SL) (including passive and interactive self ligating braces) and the conventional brackets group (CL). The power analysis showed that 16 patients should be recruited in each group.

The sample consisted of 60 subjects were randomly selected from three profession orthodontists who had the same concept of treatment philosophy and were familiar with each other (Table 1). Subjects included in the study satisfied the following selection criteria: patients must be (1) older than 12 years; (2) Hawley retainer was used in both upper and lower dental arch approximately 2 years.; (3) A non-extraction treatment plan; (4) Class I molar relationship, (5) crowding less than 5 mm (6) follow-up at least more than 5 years (6) permanent dentition (7) treatment included 0.022-in slot brackets with similar wire sequences (SL brackets, Time, Adenta, Gilching/Munich, Germany, or SmartClip,3 M Unitek, Monvoria, Calif; or CL brackets, 3 M Unitek, Monrovia, Calif,) (8) extract third molar if impacted.

**Table 1:** **Demographics and clinical characteristics of sample**

Variable	SL group		CL group		P value
	Mean	SD	Mean	SD	
Age	13.56	1.62	13.48	1.46	0.53
gender					
male	15		14		
female	15		16		0.34
Follow up period	7.24	1.32	7.68	1.6	0.46
Arch length Md	50.36	5.21	50.12	4.98	0.59
Arch length Max	59.32	4.68	59.12	4.96	0.35
Inter-canine width Md	27.36	1.40	28.12	1.34	0.36
Inter-canine width Max	34.23	1.68	34.26	1.59	0.33
Inter-molar width Md	36.26	2.13	36.59	2.36	0.48
Inter-molar width Max	42.32	2.36	41.89	2.29	0.67
II Md	11.26	4.56	11.89	5.36	0.24
II Max	10.38	3.89	9.87	4.12	0.18
PAR	28.48	10.23	27.68	10.98	0.34

Max indicates maxillary; Md indicates mandibular; II indicates Irregularity Index; P > 0.05 indicates no statistically significant change.

Patients with hypodontia, oligodontia, hypothyroidism, cleft-lip/palate, syndromes were excluded. Participants who met these inclusion criteria were recruited. At the time of recruitment, it was routine practice to obtain written consent for participation in the trial.

### Methods

Dental casts were routinely made at the following stages: pre-treatment (TP); post-treatment (T0); 2 years after T0 (T2); more than 5 years after T0 (T5). Five variables were measured, including the following: Irregularity Index [15]; Inter-canine width; Inter-molar width; PAR index [16]; arch length.

Three examiners were incorporated in this study. To determine the measurement error in the PAR and assess the intra-observer and inter-observer agreement, 18 randomly selected patients were evaluated by the three observers. The dental casts at TP and at T5 were re-measured for these patients. The time interval between two intra-observer assessments was at least 3 weeks.

### Statistical analysis

Descriptive and analytically statistical analyses were performed with SPSS software (release 18.0, SPSS for Windows). Systematic differences between observers were tested by the paired *t* test. Inter-observer and intra-observer reliability was expressed as Pearson’s correlation coefficients between re-measurements. The magnitude of the intra-observers and inter-observers measurement error in the PAR was calculated.

Statistical analysis was performed by using standard methods. Groups were compared by Student’s *t*-test for independent group, and significance of changes across time was determined by the Student’s *t*-test for paired data. The level of statistical significance was established at *P* < 0.05.

## Results

No significantly systematic differences were found between examiners. The measurement errors were 0.9. The intra-observer correlation ranged over the two periods from 0.98 to 0.99 and the inter-observer correlation from 0.96 to 0.99, indicating a high level of reliability. No significant differences were detected in age, gender, follow up period, and PAR and Little index before treatment (Table 1).

Three dimensions of the dental arch, including maxillary inter-canine width and the two inter-molar widths, did not change significantly over the long-term follow-up period for each group (Table 2).

**Table 2:** **Differences between Post-treatment and Post-retention Arch Dimensions (mm) of SL and CL group**

Variable	SL group		Cl group		P
	Mean	SD	Mean	SD	
Arch length Max					
T(0)-T(P)	1.68	1.34	1.23	1.45	0.23
T(2)-T(0)	-2.09	1.21	-1.99	1.35	0.16
T(5)-T(2)	-0.71	0.35	-0.93	0.23	0.45
Arch length Md					
T(0)-T(P)	2.31	1.56	1.98	1.76	0.36
T(2)-T(0)	-1.23	0.83	-1.29	0.79	0.48
T(5)-T(2)	-1.08	0.79	-1.10	0.86	0.38
Inter-canine width Max					
T(0)-T(P)	1.89	1.23	1.86	1.36	0.47
T(2)-T(0)	-2.79	1.45	-2.61	1.21	0.26
T(5)-T(2)	-0.34	0.78	-0.31	0.67	0.56
Inter-canine width Md					
T(0)-T(P)	0.68	1.46	0.56	1.56	0.35
T(2)-T(0)	-2.19	1.39	-1.98	1.36	0.47
T(5)-T(2)	-0.24	0.36	-0.54	0.52	0.12
Inter-molar width Max					
T(0)-T(P)	2.36	1.20	1.12	0.87	*
T(2)-T(0)	-2.13	1.35	-2.03	1.52	0.34
T(5)-T(2)	-0.78	0.32	-0.76	0.36	0.21
Inter-molar width Md					
T(0)-T(P)	2.14	1.56	2.06	1.68	0.32
T(2)-T(0)	-2.68	1.26	-2.55	1.36	0.26
T(5)-T(2)	-1.23	1.32	-1.34	1.36	0.18

Max indicates maxillary; Md indicates mandibular; T(P) = pretreatment; T(0) = post-treatment; T(2) = 2 years post-retention; T(5) = more than 5 years post-retention; P > 0.05 indicates no statistically significant change.

Inter group comparison results showed that, at T (P) and T (0), both groups presented smaller PAR and Little indexes. In the follow up period, both groups showed minor increasing in PAR and Little maxillary indexes, but the changes in irregularity index and the PAR index were not significantly greater in SL group than in CL group (Table 3).

**Table 3:** **Post-treatment and Post-retention Occlusal Dimensions (mm, except for PAR) of SL and CL group**

Variable	SL group		Cl group		P
	Mean	SD	Mean	SD	
II Max					
T(0)-T(P)	-9.56	4.68	-8.96	5.12	0.25
T(2)-T(0)	0.89	1.26	0.79	1.45	0.12
T(5)-T(2)	0.68	1.11	0.56	1.34	0.09
II Md					
T(0)-T(P)	-10.68	5.23	-10.69	5.69	0.35
T(2)-T(0)	1.69	1.23	1.89	1.35	0.25
T(5)-T(2)	0.26	0.78	0.34	0.66	0.39
PAR					
T(0)-T(P)	-26.68	10.26	-25.88	10.98	0.78
T(2)-T(0)	1.64	1.98	1.56	1.68	0.35
T(5)-T(2)	0.98	1.25	1.23	1.39	0.37

Max indicates maxillary; Md indicates mandibular; II indicates Irregularity Index; T(P) = pretreatment; T(0) = post-treatment; T(2) = 2 years post-retention; T(5) = more than 5 years post-retention; P > 0.05 indicates no statistically significant change;* indicates statistically significant change.

## Discussion

The orthodontists’ goal for the patients is to have a satisfactory occlusion and alignment of the teeth after many years post-retention [17]. However, relapse is an inevitable outcome of combined action of many factors, so how to maintain the stability of teeth become urgent problems to orthodontists.

The results of this study indicate a satisfactory long-term post-retention stability, as defined by Little’s irregularity index of <3.5 mm, which is achievable in both groups. These findings agree with previous studies which indicated a satisfactory post-retention stability (irregularity index <3.5 mm) for their samples or subsamples [18–21].

It is generally agreed that dental arch form and width should be maintained during orthodontic treatment [22, 23]. Several studies showed that inter-canine and inter-molar widths decreased during the post-retention period, especially if it had been expanded during treatment [24–27]. For this reason, the maintenance of arch form is generally recommended.

This study found that the dental arch had a certain degree expansion in both groups and that there was no statistically significant increases in inter-canine width; but SL brackets resulted in statistically greater increase in inter-molar width than conventional appliances. However SL did not show greater post-retention decrease in their molar width than CL, which may be related to the fact that the two groups existed statistically significant differences, but the difference was only 1 mm, which was not clinically different. A systematic review [28] found the similar result that arch dimensional changes arising with SLBs and conventional systems appeared to be similar: identical levels of incisor proclination and inter-canine expansion developed in both systems.

In this study, the mean reduction in the PAR at the end of active treatment was 26.68 ± 10.26 in SL group, while 25.88 ± 10.98 for CL group. It means that PAR score reductions were unaffected by the choice of appliances. After more than 5 years post-retention, PAR score slightly increased in both groups, but the increase was still no significantly different. This indicated that most of the achieved orthodontic treatment results had been maintained in both groups after more than 5 years post-retention. This revealed that small amount of relapse is not only the result of orthodontic treatment, but also due to the physiological and pathological changes in the dentition and surrounding tissues during those years. It had been shown by Behrents [29] and Schols et.al [30] that considerable craniofacial alteration occurred beyond the age of 17 years old in human beings. This process was accompanied by compensatory changes in the dentition. The orthodontist has little control over these biological processes.

The claim of reduced friction with self-ligating brackets was often cited as a primary advantage over conventional brackets [31]. With reduced friction and hence less force was needed to produce tooth movement [32], self-ligating brackets are proposed to have the potential advantages of producing more physiologically harmonious tooth movement not by overpowering the musculature and interrupting the periodontal vascular supply [33].

Previous studies also stated that moderate crowding was alleviated about 2.7 times faster with Damon 2 brackets than with conventional appliances [34]. Therefore, it may made CL group easier to recurrence, but the results found in our study indicated that there was no statically significant increases of irregular index in both groups. The reason of recurrence may be the result of comprehensive factors work together. As previously reported, post-retention increases in irregularity were not correlated with crowding before treatment or amount of treatment change [35]. There was a tendency for the mandibular incisor to rebound, These findings could be interpreted to support Blake’s [36] contention that the initial position of the mandibular incisors is the best guide for their lab-lingual stable position and many investigators who had noted a rebound effect for displaced incisors [37–39].

A limitation of this study was designed as a longitudinal retrospective study. Generally speaking, it is very difficult to avoid confounding factors. But, we can reduce the effects of bias by making strict inclusion criteria. AS shown in Table 1, we can see the baseline levels of two groups are in consistency. And malocclusion type might affect the results, therefore, in this study only patients with Angle Class I were included so as to minimize the impact of confounding factors on the experimental results. Another problem related to longitudinal retrospective studies might be the information bias. We had gone through repeated trials, and surveyors and final statistics do not know the group of data. Therefore, this study was relatively real, which may reflect the actual results accurately.

PAR, Little irregularity index, dental arch length and width index measured in our study can comprehensively reflect the statues of relapse, because relapse not only included the change of overbite/overjet, but also the change of dental arch length and width. Some researches [1, 17] used only PAR or irregular index to evaluate long term stability, which has certain one-sidedness.

In a word, in terms of long term stability between SL and CL brackets, no significant differences were found in our study. Due to the fact that self-ligating braces are expensive, the advantages of saving time and reducing root absorption still need stronger evidence to be proven, meanwhile, given that cost-effectiveness, orthodontists should consider multiple factors in choosing suitable orthodontic bracket type for the patients.

## Conclusions

-  There were no statistical differences in long-term stability of treatment between self-ligating brackets and conventional brackets.

## References

[1] Al Yami EA, Kuijpers-Jagtman AM, Van’t Hof MA (1999). Stability of orthodontic treatment outcome: follow-up until 10 years post-retention. Am J Orthod Dentofacial Orthop.

[2] Elms TN, Buschang PH, Alexander RG (1996). Long-term stability of Class II, Division 1, nonextraction cervical face bow therapy: I. model analysis. Am J Orthod Dentofacial Orthop.

[3] Elms TN, Buschang PH, Alexander RG (1996). Long-term stability of Class II, Division 1, nonextraction cervical face bow therapy: II. cephalometric analysis. Am J Orthod Dentofacial Orthop.

[4] Rossouw PE, Preston CB, Lombard CJ, Truter JW (1993). A longitudinal evaluation of the anterior border of the dentition. Am J Orthod Dentofacial Orthop.

[5] Fudalej P, Artun J (2007). Mandibular Growth Rotation Effects on Postretention Stability of Mandibular Incisor Alignment. Angle Orthod.

[6] Harradine NW, Pearson MH, Toth B (1998). The effect of extraction of third molars on late lower incisor crowding: a randomized controlled trial. Br J Orthod.

[7] Edwards JG (1988). A long-term prospective evaluation of the circumferential supracrestal fiberotomy in alleviating orthodontic relapse. Am J Orthod Dentofacial Orthop.

[8] Little RM, Riedel RA, Engst ED (1990). Serial extraction of first premolars—postretention evaluation of stability and relapse. Angle Orthod.

[9] Chen SS, Greenlee GM, Kim JE, Smith CL, Huang GJ (2010). Systematic review of self-ligating brackets. Am J Orthod Dentofacial Orthop.

[10] Harradine NW (2003). Self-ligating brackets: where are we now?. J orthod.

[11] Henao SP, Kusy RP (2004). Evaluation of the frictional resistance of conventional and self-ligating bracket designs using standardized archwires and dental typodonts. Angle Orthod.

[12] Turnbull NR, Birnie DJ (2007). Treatment efficiency of conventional vs self-ligating brackets: effects of archwire size and material. Am J Orthod Dentofacial Orthop.

[13] Eberting JJ, Straja SR, Tuncay OC (2001). Treatment time, outcome, and patient satisfaction comparisons of Damon and conventional brackets. Clin Orthod Res.

[14] Čelar A, Schedlberger M, Dörfler P, Bertl M (2013). Systematic review on self-ligating vs. conventional brackets: initial pain, number of visits, treatment time. J Orofac Orthop.

[15] Little RM (1975). The irregularity index: a quantitative score of mandibular anterior alignment. Am J Orthod.

[16] Richmond S, Shaw WC, Roberts CT, Andrews M (1992). The PAR index (Peer Assessment Rating): methods to determine outcome of orthodontic treatments in terms of improvement and standards. Eur J Orthod.

[17] Boley JC, Mark JA, Sachdeva RC, Buschang PH (2003). Long-term stability of Class I premolar extraction treatment. Am J Orthod Dentofacial Orthop.

[18] Dugoni SA, Lee JS, Varela J, Dugoni AA (1995). Early mixed dentition treatment: postretention evaluation of stability and relapse. Angle Orthod.

[19] Sadowsky C, Schneider BJ, BeGole EA, Tahir E (1994). Long-term stability after orthodontic treatment: nonextraction with prolonged retention. Am J Orthod Dentofacial Orthop.

[20] Vaden JL, Harris EF, Gardner RL (1997). Relapse revisited. Am J Orthod Dentofacial Orthop.

[21] Glenn G, Sinclair PM, Alexander RG (1987). Nonextraction orthodontic therapy: posttreatment dental and skeletal stability. Am J Orthod Dentofacial Orthop.

[22] McCauley DR (1944). The cuspid and its function in retention. Am J Orthod.

[23] Riedel RA (1960). A review of the retention problem. Angle Orthod.

[24] Welch KN (1956). A study of treatment and postretention dimensional changes in mandibular dental arches [MSD Thesis].

[25] Amott RD (1962). A serial study of dental arch measurements on orthodontic subjects: 55 cases at least 4 years postretention [MSD Thesis].

[26] Arnold ML (1963). A study of the changes of the mandibular intercanine and intermolar widths during orthodontic treatment and following postretention period of five or more years [MSD Thesis].

[27] Kahl-Nieke B, Fischbach H, Schwarze CW (1995). Post-retention crowding and incisor irregularity: a long-term follow-up evaluation of stability and relapse. Br J Orthod.

[28] Fleming PS, Johal A (2010). Self-ligating brackets in orthodontics. A systematic review. Angle Orthod.

[29] Behrents RG (1985). Growth in the aging craniofacial skeleton. Monograph 17. Craniofacial Growth Series.

[30] Schols JGJH (1988). Van der Linden FPGM:Development of the dentition and facial growth during adolescence. Inform Orthod Kieferorthop.

[31] Kim TK, Kim KD, Baek SH (2008). Comparison of frictional forces during the initial leveling stage in various combinations of self-ligating brackets and archwires with a custom-designed typodont system. Am J Orthod Dentofacial Orthop.

[32] Berger JL (1990). The influence of the SPEED bracket’s self-ligating design on force levels in tooth movement: a comparative in vitro study. Am J Orthod Dentofacial Orthop.

[33] Damon DH (1998). The rationale, evolution and clinical application of the self-ligating bracket. Clin Orthod Res.

[34] Pandis N, Polychronopoulou A, Eliades T (2007). Self-ligating vs conventional brackets in the treatment of mandibular crowding: A prospective clinical trial of treatment duration and dental effects. Am J Orthod Dentofacial Orthop.

[35] Little RM, Riedel RA, Stein A (1990). Mandibular arch length increase in the mixed dentition: postretention evaluation of stability and relapse. Am J Orthod Dentofacial Orthop.

[36] Blake M, Bibby K (1998). Retention and stability: a review of the literature. Am J Orthod Dentofacial Orthop.

[37] Weinstein S, Haack DC, Morris LY, Snyder BB, Attaway HE (1963). On an equilibrium theory of tooth position. Angle Orthod.

[38] Brodie AG (1945). Does scientific investigation support the extraction of teeth in orthodontic therapy?. Am J Orthod Oral Surg.

[39] Litowitz R (1948). A study of the movements of certain teeth during and following orthodontic treatment. Angle Orthod.

